# Tubal Cancer Clinical Management: Two Exceptional Scenarios and a Review of the Literature

**DOI:** 10.3390/jcm13175075

**Published:** 2024-08-27

**Authors:** Irene Colombi, Marco D’Indinosante, Lucia Lazzeri, Errico Zupi, Silvia Pisaneschi, Marco Giusti, Alberto Mattei, Elisa Valentina Debonis, Angelo Cassisa, Anna Franca Cavaliere, Federica Perelli

**Affiliations:** 1 Department of Molecular and Developmental Medicine, Obstetrics and Gynecological Clinic, University of Siena, 53100 Siena, Italy; colombi.irene1@gmail.com (I.C.); lucialazzeri79@gmail.com (L.L.); errico.zupi@unisi.it (E.Z.); 2 Department of Women’s, Children’s and Public Health Sciences, Fondazione Policlinico Universitario Agostino Gemelli IRCCS, 00168 Rome, Italy; marco.dindinosante@gmail.com; 3 Department of Gynecology and Pediatrics, Azienda USL Toscana Centro, 50100 Florence, Italy; silvia.pisaneschi@uslcentro.toscana.it (S.P.); marco1.giusti@uslcentro.toscana.it (M.G.); alberto.mattei@uslcentro.toscana.it (A.M.); 4 Department of Oncology, Section of Pathology, San Giovanni di Dio Hospital, USL Toscana Centro, 50143 Florence, Italy; elisavalentina.debonis@uslcentro.toscana.it (E.V.D.); angelo.cassisa@uslcentro.toscana.it (A.C.); 5 Division of Gynecology and Obstetrics, Isola Tiberina Gemelli Hospital, 00186 Rome, Italy; annafranca.cavaliere@fbf-isola.it

**Keywords:** tubal cancer, ovarian cancer, tubal carcinoma, primary debulking surgery, serous tubal intraepithelial carcinoma, STIC, high-grade serous carcinoma

## Abstract

This article provides a literature review on tubal carcinoma to offer an updated insight into its preventative strategies, diagnosis, treatment and oncological surveillance. In addition to the search string utilized, the authors’ focus extended to key scientific studies, consensus statements, guidelines and relevant case reports essential for the proper clinical management of the disease, providing a methodologically well-structured literature review combined with practical expertise in the oncological field. This article also includes two special clinical cases that emphasize the importance of understanding the physiopathology and the current state of the art in the anatomopathological advancements in tubal/ovarian/peritoneal carcinoma, often assimilated into a single clinical entity and to which many of the concepts extracted from the literature can apply.

## 1. Introduction

The fallopian tubes are one of the least affected organs in gynecological cancers, accounting for less than 2% of all gynecological malignancies [1,2]. Tubal precancerous lesions play a crucial role in the development of ovarian high-grade serous carcinoma (HGSC), making close clinical monitoring of these pathologies necessary [3]. HGSC is one of the most aggressive ovarian cancers, with a 5-year survival rate of 49% [4], and is responsible for the majority of deaths from ovarian malignant neoplasms, mainly because it is usually diagnosed at an advanced stage. It generally affects postmenopausal women, and has been widely associated with the breast cancer (BRCA) 1–2 gene mutation, with an incidence of 16–44% in this population [5]. Another theory for the development of fallopian tube cancer is that substances released into the pelvic cavity during ovulation contribute to DNA damage to the fallopian tube cells.

A strong association has been found between HGSC and a tubal lesions considered to be a precursor, known as serous tubal intraepithelial carcinoma (STIC) [6].

Tubal cancer and serous ovarian and primary peritoneal carcinomas share overlapping ultrasonographic features, treatment strategies and prognoses, and therefore, in most guidelines [7,8] these three entities are considered jointly.

The typical appearance of a tubal carcinoma on ultrasound is a dilated tube, some-times containing highly vascularized solid tissue. However, tubal abscess, endometriosis, tuberculosis and other non-oncological conditions can be mistaken for tubal neoplasia due to their similar features and require evaluation by an experienced operator. In addition, tubal carcinoma can often be misdiagnosed and confused either with a benign pathology due to overlapping ultrasonographic features or with a primary ovarian tumor as a result of the fallopian tube being partially or completely involved in the tubo-ovarian mass.

Tubal cancer is usually staged surgically and, according to the Fédération Interna-tionale de Gynécologie et d’Obstétrique (FIGO), can be classified into early and advanced stages from stages I to IV [7]. Tubal stage I lesions are confined to one or both tubes, while extension beyond the tube with involvement of the pelvic organs is classified as stage II. Stage III is recognized as the presence of extension beyond the pelvis, and stage IV as the presence of distant metastases. However, tubal cancer is considerably rarer than ovarian cancer, and the aim of this review is to discuss the diagnostic, therapeutic and prognostic aspects of tubal cancer to raise awareness of this disease which, although rare, deserves special attention.

## 2. Materials and Methods

A search for relevant articles was conducted in PubMed from July 2014 to July 2024. The Search String was “tubal cancer AND surgery”. The inclusion criteria were as follows: 

The studies included in the review were defined by population, intervention, comparison, outcomes and study design (PICOs), and we selected studies about women affected by tubal cancer submitted to surgery comparing survival outcomes; only articles written in English were considered; and only studies published within the time frame relevant to the research question were included in the review (from July 2014 to July 2024).

The exclusion criteria were as follows:

Studies that did not meet the defined inclusion criteria; duplicated studies; non-peer-reviewed articles, gray literature, or reports lacking scientific rigor; and meta-analyses, reviews and systematic reviews.

We found 644 publications, 1 of which was excluded because the full text was not available. After excluding meta-analyses, reviews and systematic reviews, a total of 24 publications remained to be included in our review. All titles and abstracts were carefully assessed, and 13 trials were finally excluded because they did not focus on the topic of the current review. The process followed the recommendations of the Preferred Reporting Items for Systematic Reviews and Meta-Analyses (PRISMA). The protocol has not been registered.

In order to provide an accurate description of the background to tubal precancerous lesions, possible progression to invasive cancer, imaging diagnosis, anatomopathological diagnosis, and staging, a further electronic search was carried out using the MEDLINE online medical database (accessed via PubMed) to evaluate the existing literature on this topic. The following terms were used in our literature search: serous intraepithelial carcinoma or STIC, high-grade serous intraepithelial tubal neoplasia, isolated serous intraepithelial tubal carcinoma, prophylactic salpingo-oophorectomy, tubal cancer and BRCA mutation. The titles and abstracts of the articles were carefully examined to select those relevant to our research question. We also conducted a thorough review of the bibliographies of the selected articles to identify additional papers for inclusion. 

All selected articles were carefully assessed for relevance and scientific merit by two independent reviewers (I.C. and F.P.). 

**Figure 1:**
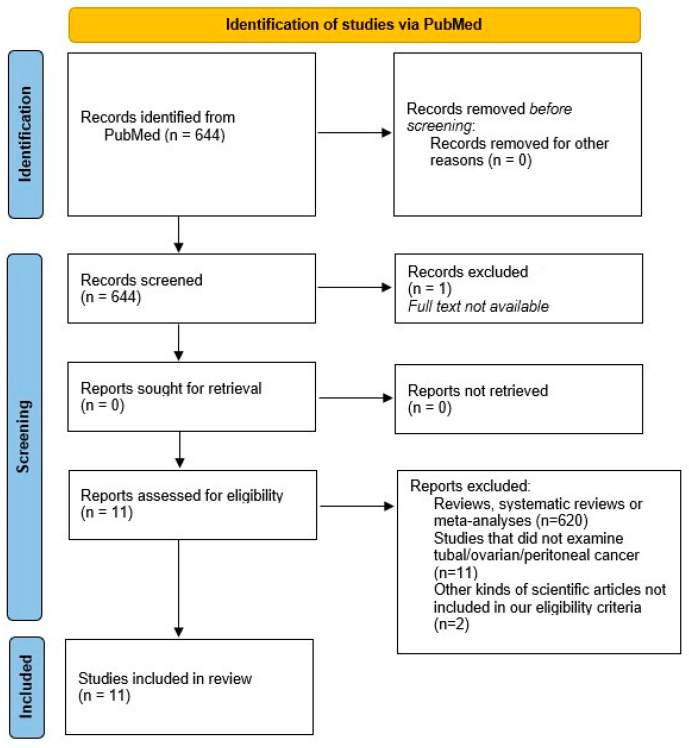
PRISMA 2020 flow diagram which includes searches of PubMed. Literature search diagram. A total of 644 papers filled the search string. Of these, 1 article was excluded because the full text was not available. In addition, 620 were excluded because they were meta-analyses, reviews or systematic reviews, so only books and documents, clinical trials and controlled trials were included. A total of 24 papers were eligible for review. After evaluating the titles and abstracts, 13 articles were excluded because they were not relevant to the topic of the review. A further 20 articles were included to provide background after searching for key words and citations [9].

A total of 31 articles were included for the purposes of our narrative review, i.e., a state-of-the-art review of knowledge on fallopian tube cancer. Figure 1 shows a literature search flow diagram. Eleven articles were selected for the review (Table 1).

## 3. Results

### 3.1. Tubal Cancer: From Precancerous Lesion to Invasive Carcinoma

The development of high-grade serous ovarian cancer from the tubal epithelium has been widely described in the literature.

One of the most accepted theories on the pathogenesis of serous ovarian cancer suggests that it may originate from a precancerous tubal lesion known as serous tubal intraepithelial carcinoma, referred to as STIC [21], which may progress into invasive carcinoma of the fallopian tube or implant on the ovarian surface, leading to the development of ovarian carcinoma [22]. 

During ovulation, the fluid ruptured from the follicles leads to the release of free radicals, reactive oxygen species and other genotoxic substances that contribute to the carcinogenesis process through DNA damage and the consequent acquisition of somatic mutations and epigenetic alterations by the tubal epithelium, as well as clonal expansion. This process appears to be accelerated in the presence of germline mutations and the epigenetic inactivation of genes such as BRCA1 and BRCA2. 

In support of this hypothesis, tubal sterilization has been associated with a reduced risk of ovarian cancer, as well as bilateral salpingo-oophorectomy, both in women with and without a BRCA mutation [21]. On the other hand, some authors postulate that STIC can theoretically spread before salpingo-oophorectomy, explaining why peritoneal HGSC can develop even after salpingectomy [23].

STIC is a rare finding, occurring in <0.1% of the general population [24] and approximately 2.3% of women at high risk of HGSC [6]. Although rare, given its potential for evolutionary malignancy, in several centers, prophylactic salpingectomy is also performed during pelvic surgery for benign pathologies, even in low-risk patients, leaving the ovaries in situ, to preserve hormonal function, and reduces the risk of future development of ovarian cancer.

Even in low-risk women undergoing surgery for benign pathologies, the fimbriated end of the tube must be carefully examined by the anatomopathologist, as occult tubal cancer may be present. If STIC is detected in the fimbriated tube, the entire organ must be analyzed as invasive carcinoma may be present [25]. For this reason, complete tubal specimen collection with detailed Sectioning and Extensively Examining the FIMbriated End (SEE-FIM) protocols are recommended [7]. However, the clinical significance and management of those patients diagnosed with STIC but at low risk of developing ovarian neoplasia remains controversial.

In the literature, the progression of STIC in serous ovarian carcinoma is reported in 10% [26] of women at high risk, although it is still not possible to conclude whether disease progression is absolutely related to STIC or if it is a de novo lesion. In addition, intraepithelial tubal metastasis may be indistinguishable from STIC [7].

Tubal proliferation can sometimes be difficult to interpret as it may not be consistent with a diagnosis of STIC. Some tubal or mucosal proliferations may be atypical without showing features of intraepithelial carcinoma. These lesions, sometimes referred to as early serous proliferations (ESP), may be diagnosed as a serous intraepithelial lesion of the fallopian tube (STIL) or a tubal intraepithelial lesion in transition (TILT), but even in these cases, their management has not been defined.

A possible evaluation for BRCA mutation has been suggested to identify women at higher risk, but data are still insufficient [25]. 

In a recent review, Patrono et al. [27] found that isolated STIC in patients with BRCA mutations developed into primary peritoneal cancer in 4.5% of cases, underlining the need for appropriate follow-up to detect recurrence at an early stage.

Nevertheless, there are insufficient data to determine the most appropriate follow-up for women with an incidental diagnosis of STIC, and the use of Cancer Antigen 125 (CA125) sampling or pelvic ultrasound remains controversial [27]. 

In a recent review, Steenbeek et al. [28] highlighted that in patients with mutated BRCA, the risk of subsequent HGSC at 5 years is 10.5% and at 10 years is 27.5%, which is significantly higher than the risk of HGSC after adnexectomy in patients without STIC findings, reported in this case to be 0.3% at 5 years and 0.9% at 10 years.

Although the potential for the malignant progression of STIC is known, there is still a lack of data on the management of these women. A German survey [29] found that as the progression of STIC to invasive cancer is estimated to be around 7 years, follow-up is generally prolonged and usually consists of annual transvaginal ultrasound (TVS) and associated serum CA125. In the case of isolated STIC, some centers propose BRCA mutation testing and laparoscopic staging surgery, although data are lacking. In addition, some centers suggest ipsilateral oophorectomy in premenopausal women and bilateral oophorectomy in menopausal women, while adjuvant chemotherapy is not generally recommended.

The National Comprehensive Cancer Network (NCCN) guidelines, updated in 2024 [8], suggest follow-up with or without CA-125 testing in the absence of invasive cancer, and surgical staging with surveillance or chemotherapy if invasive cancer is present in specimens. 

As with other guidelines, the NCCN also agree that genetic counseling and testing should be performed if not previously conducted in the event of STIC, but they also agree that the beneficial role of surgical staging and/or adjuvant chemotherapy in the incidental finding of STIC is still being debated.

An accurate study of the STIC entity is essential in future prospectives to ascertain whether to perform only salpingectomy in premenopausal women at high risk of HGSC and deepening ovariectomy after menopause [5].

### 3.2. Imaging Diagnosis

The diagnosis of both primary peritoneal cancer and fallopian tube cancer is generally postoperative [8], although the literature describes pathological aspects on imaging modalities of the fallopian tubes. 

Affected fallopian tubes appear macroscopically as enlarged structures with irregular inner walls due to the presence of solid tissue protruding into the lumen. Hemorrhage and necrosis are very common [2]. Ludovisi et al. [2] described the main ultrasonographic features of tubal carcinoma. In most cases, the affected tube shows a cystic, sausage-like appearance. In contrast to acute inflammation, it presents thin walls with an irregular inner surface due to the presence of solid tissue and papillary projections. On a power Doppler, the walls and solid tissue are strongly vascularized. Tubal carcinoma may present alternatively, as a tubal structure with a voluminous solid component or as a completely solid mass with no fluid content. If present, the inner cystic fluid is usually anechoic, and the affected tube may be erroneously misdiagnosed as a hydrosalpinx.

In flogistic pathology, the appearance of the fallopian tubes is quite different and may be confusing to the untrained observer. Acutely infected tubes often have a “cogwheel” appearance in transverse sections due to the edematous inner wall protruding into the lumen, whereas in longitudinal sections, the same walls indicate the presence of incomplete septa. These septa are usually highly vascularized. In chronic salpingitis, the internal protrusions are thinner and delineate a characteristic sign called “beard on a string”, the tube appears elongated, and the walls are thinner and less vascularized. These sonographic aspects overlap with those of tubal cancer and make differential diagnosis difficult. If the tubal mass is carefully examined and the internal protrusion within the lumen is composed of a solid component, the fluid content in acute flogosis is usually like ground glass due to the presence of pus in the tube, and the tubal walls are thicker. For this reason, the lesion must be examined both longitudinally and transversely, and in the latter case, the presence of incomplete septa or a cogwheel appearance tilts the balance towards a benign diagnosis.

Tongsong et al. [30] confirmed these ultrasound patterns, stating that in their series, the affected tubes also showed a sausage-shaped structure on TVS with solid tissue protruding into it. In contrast to benign tubal pathologies, incomplete septa were present in only 33.3% of cases. In 40% of patients, the ovaries appeared normal. 

In their series, they also reported a few cases that were misdiagnosed as tubal carcinoma preoperatively but were found to be ovarian carcinoma after surgery. According to Tongsong et al., the misdiagnosis was due to the presence of an ovarian cystic structure with overlapping characteristics with tubal pathology, mimicking the sausage-shaped aspect. In 62% of cases, tubal cancer is an incidental finding in asymptomatic women and is often discovered at an advanced stage [2]. Tubal cancer can be predicted on TVS by applying simple International Ovarian Tumor Analysis (IOTA) rules with pattern recognition, with a sensitivity of 86.7% and a specificity of 97.4% [30]. 

In the presence of an adnexal cyst, it is essential to search carefully for the ipsilateral ovary. In the case of a malignant lesion, it is important to distinguish between an ovarian or a tubal origin, but in difficult cases, this is not a primary concern, as these pathologies share the same classification for staging, treatment and prognosis [2].

Magnetic resonance imaging (MRI) is a second-line imaging technique often used in the preoperative staging of gynecological diseases. Tubal carcinomas appear on MR as a hyperintense T2-weighted signal and a hypointense T1-weighted signal showing a solid aspect [31].

The actual staging system for ovarian, fallopian tube and peritoneal cancer according to the FIGO [32] classification should be based on the surgical findings of primary debulking surgery (PDS). Nevertheless, some authors argue that the imaging modalities computed tomography (CT) and magnetic resonance can also be used to recognize the origin of the lesions and tumor stage when combined with cytological examination and tumor marker levels without resorting to diagnostic surgery in advanced stages [16].

### 3.3. Anatomopathological Diagnosis

The diagnosis of tubal pathology can be challenging, particularly in the case of incidental pre-cancerous lesions. To provide a standardized, reproducible diagnosis, the Sectioning and Extensively Examining the FIMbri-ated End (SEE-FIM) protocol has been developed. The SEE-FIM procedure consists of embedding the entire fallopian tube with an explicit focus on the fimbriated end [24]. Nevertheless, the SEE-FIM protocol cannot be applied by default, given the low probability of finding an STIC lesion in the general population, and the fact that STIC has no clinical consequence when diagnosed concomitantly with HGSC. Therefore, this protocol should be used in selected cases, in women at high risk or in the presence of atypical findings on initial anatomopathological evaluation [24].

In order to standardize the diagnosis of STIC, Bogaerts et al. [24], based on a consensus statement, developed a few recommendations to describe the diagnostic workup that should be performed by a pathologist. The proposed process is divided into five domains, including processing and macroscopy, microscopy, immunohistochemistry, interpretation and reporting and miscellaneous. Consensus was reached through a Delphi study involving 34 expert pathologists from 11 countries worldwide.

The examination starts from a slide at low magnification, with a maximum of 5 times magnification, looking for areas of cytological atypia which, if present, must be examined at higher magnification, with the aim of recognizing the distinctive morphological features proposed by the consensus for the diagnosis of STIC. 

The characteristic cytological changes are nuclear pleomorphism, nuclear enlargement, high nuclear/cytoplasmic ratio and nuclear hyperchromasia. 

The second step is an evaluation of immunohistochemistry, which is performed in all cases of atypical morphology, especially p53 and Ki67. An abnormal p53 must always be found to establish a diagnosis of STIC, while for Ki67, the data are confused, and a proliferation index higher than 10% is considered abnormal, even if the cut-off values are still unclear.

Diagnosis is more difficult in lesions that do not meet the diagnostic criteria for STIC, for example in so-called p53 signatures, characterized by an aberrant p53 staining pattern in at least 12 adjacent cells but no clear cytomorphological atypia on hematoxylin and eosin staining, or in STIL and TILT, which resemble STIC but where immunohistochemical staining for p53 and Ki-67 does not fully support the diagnosis. Secretory or stem cell outgrowths (SCOUTs) are a group of proliferative lesions in the fallopian tube epithelium that are not associated with p53 mutation but show overlapping cytological changes that make diagnosis difficult [24] (Table 2).

HGSC is an inhomogeneous disease with different histological features, coexisting with STIC, occurring at different ages, and showing different clinical outcomes [33].

The histological features of HGSC classically show a papillary, micropapillary or infiltrative pattern in more than 50% of tumors, often with a desmoplastic stroma [33].

### 3.4. Staging and Management

Fallopian tube, primary peritoneal and ovarian cancer are considered together in almost all major guidelines because of their common diagnosis, treatment and prognosis, especially in the case of HGSC, where the pathogenesis is also shared. 

A standardized diagnosis of STIC is essential. One of the main reasons is that it has prognostic implications related to an increased risk of peritoneal carcinomatosis, which opens the debate about the usefulness of surgical staging or chemotherapy in women with isolated and incidental STIC. In any case, there is consensus among clinicians that the findings of STIC require follow-up strategies for the prevention of tubo-ovarian or peritoneal cancer [24].

According to the NCCN guidelines [8], follow-up options include observation alone with or without CA-125 testing if there is no evidence of invasive cancer, but it is still not clear whether surgical staging and/or adjuvant chemotherapy would be beneficial. There is consensus that genetic evaluation is mandatory in women with incidental findings of STIC.

On the other hand, in the case of invasive cancer, surgical staging with the observation or chemotherapy is usually required. 

For surgical staging, minimally invasive surgery is generally the first choice to assess the extent of the disease, to allow an eventual diagnosis through frozen section examination or to decide, in cases with peritoneal carcinomatosis, on the need to perform PDS or interval debulking surgery (IDS) after neoadjuvant chemotherapy (NACT) [34]. The upper abdomen, bowel surfaces, omentum, appendix and pelvic organs should be carefully examined, and abnormal findings should be biopsied and cytology obtained by peritoneal washings. If cytoreduction is possible, a total bilateral salpingo-oophorectomy is required, as well as removal of 2 cm of the infundibolo-pelvic ligament and the peritoneum surrounding the ovaries and fallopian tubes. To avoid traumatic exfoliation of the cells, an endobag should always be used to retrieve the specimens.

Hysterectomy should be considered in women with an increased risk of endometrial cancer, BRCA1 mutations, Lynch syndrome or exposure to tamoxifen.

Recognizing the primary origin of HGSC is fundamental. It should only be classified as ovarian in origin if both tubes appear normal on macroscopic examination and the SEE-FIM protocol. The staging is IIA tubal HGSC if STIC and ovarian HGSC are present simultaneously.

Synchronous independent neoplasms are very rare; in the presence of lesions in both the ovary or fallopian tubes and the endometrium, they should be considered as metastases from one of these sites.

The 2024 NCCN guidelines [8] recommend the following elements to be evaluated for staging: the location and size of lesions, presence or absence of surface involvement, integrity of the specimen; histological type and grading; presence of implants that are a barrier to biopsy; cytology of fluid collected; evaluation of lymph nodes examined; and evidence of STIC, endometriosis and endosalpingiosis.

The treatment of primary peritoneal and fallopian tube cancer is the same as the treatment of epithelial ovarian cancer, and it usually consists of surgical staging and debulking surgery, which may be followed by systemic chemotherapy. However, in advanced stages, when primary debulking surgery is not possible due to advanced age, frailty, poor performance status, co-morbidities, or an inability to perform cytoreduction, NACT with IDS should be considered. In early-stage disease, instead, surgery alone and close follow-up may be enough [8]. 

Debulking surgery for suspected malignant ovarian, fallopian tube or primary peritoneal neoplasm should be performed by open laparotomy with a vertical midline abdominal incision; minimally invasive techniques may be an option only in early-stage disease or when the surgeon judges that optimal debulking, i.e., residual disease less than 1 cm, can be achieved in selected patients after NACT.

### 3.5. Preventive Management and Fertility-Sparing Options

Preventive strategies for tubal cancer, particularly for women at high risk due to genetic predispositions such as BRCA1 or BRCA2 mutations, often focus on risk-reducing salpingo-oophorectomy (RRSO). This surgical procedure involves the removal of the ovaries and fallopian tubes and can significantly decrease the incidence of tubal and ovarian cancer.

The process typically begins with genetic counseling for women who may have a family history of ovarian or breast cancer. This counseling helps assess their risk and may lead to genetic testing for BRCA mutations, which plays a crucial role in informing decisions regarding preventive measures. A comprehensive evaluation of personal and family medical history is essential to determine an individual’s risk of developing tubal and ovarian cancers, guiding the recommendation for RRSO. 

Timing is another critical aspect; for women identified as being at genetic risk, RRSO is generally recommended after they have completed childbearing, typically between the ages of 35 and 40, although the specific timing may vary based on individual circumstances and additional risk factors. Before proceeding with RRSO, healthcare providers may suggest regular screening for cancers through methods such as TVS and CA125 blood tests to monitor for any initial signs of cancer, especially for women who choose to delay surgery.

Education is vital, as women should be made aware of the signs and symptoms of tubal and ovarian cancer. Understanding the importance of consulting healthcare providers regarding any concerning changes can lead to earlier interventions. A comprehensive patient care approach is also beneficial, involving gynecologists, oncologists, and mental health professionals to provide support throughout the decision-making process and after RRSO, addressing both physical and emotional health needs.

Following RRSO, women will experience hormonal changes due to the removal of their ovaries, making discussions about hormone replacement therapy (HRT) important for managing symptoms and reducing long-term health risks associated with early menopause. Additionally, encouraging healthy lifestyle choices, such as following a balanced diet, engaging in regular exercise, and maintaining a healthy weight, can positively contribute to overall health and may help lower cancer risk.

RRSO, combined with genetic counseling, thorough risk assessment, and personalized healthcare support, serves as a cornerstone of preventive strategies for women at high risk of tubal cancer. By tailoring the approach to each individual, healthcare providers can enhance the effectiveness of these preventive measures.

Fertility sparing may be considered in selected patients who wish to achieve pregnancy and who appear to have unilateral stage IA tumors, or in those with bilateral stage IB tumors where the uterus is preserved.

Fertility-sparing treatment options for tubal cancer are focused on preserving a woman’s ability to conceive while effectively managing the cancer. Certain criteria help determine if a patient is suitable for fertility-sparing approaches.

First and foremost, the stage of cancer is critical; fertility-sparing options are generally considered for patients with early-stage disease, particularly those diagnosed with stage I cancer, where the tumor is confined to the fallopian tube and has not spread. Specifically, non-invasive tumors or very early invasive tumors may also be eligible for these approaches.

Additionally, the type of tumor plays a significant role. Tumors that are low-grade serous carcinomas, which exhibit a better prognosis, tend to be more amenable to fertility-sparing interventions. Furthermore, the aggressiveness of the tumor is a key factor; well-differentiated tumors are often more suitable for such treatments.

The age of the patient is another important criterion, with younger women, typically under the age of 40, being prioritized for fertility-sparing options. The personal desire for future fertility is also taken into account, as it is crucial to align treatment decisions with the patient’s wishes regarding having children in the future.

The overall health status of the patient should not present significant comorbidities that could complicate treatment or future pregnancies. A recommendation from a multidisciplinary team, including oncologists, gynecologists, and fertility specialists, is essential. This collaborative evaluation ensures that the chosen fertility-sparing approach does not compromise effective cancer treatment and outcomes in terms of disease-free and overall survival.

Informed consent and counseling are imperative in this decision-making process. Patients should be thoroughly counseled about the risks and benefits of fertility-sparing treatments, including any potential impacts on cancer prognosis and future pregnancies. Lastly, a clear follow-up plan that may involve surveillance and potential adjuvant therapies after fertility-sparing surgery is necessary to monitor health outcomes.

Ultimately, each case is assessed on an individual basis, considering the specific diagnosis and personal circumstances of the patient. Options such as salpingectomy (removal of the fallopian tube) or salpingo-oophorectomy (removal of the tube and ovary) with careful follow-up can serve the dual purpose of addressing cancer while preserving reproductive function. Collaboration with fertility specialists is also vital, particularly for discussing options like ovarian tissue cryoconservation, egg freezing or embryo preservation, if engaged in future family planning. 

In recent years, a growing number of women have chosen to postpone childbearing until their late 30s and even into their 40s. This shift is influenced by various social, economic and personal factors. Many women prioritize education and career development, seeking to establish professional stability before starting a family. Additionally, advances in reproductive technology, such as in vitro fertilization (IVF) and egg freezing, have made it increasingly feasible for women to conceive later in life. 

While this trend affords women greater control over their reproductive choices, it also presents certain challenges. As women age, the risks associated with pregnancy, including complications such as gestational diabetes, hypertension, and chromosomal abnormalities, tend to increase. Fertility declines with age, leading to a higher likelihood of infertility and the need for assisted reproductive technologies. 

However, with the increasing age at which women are becoming pregnant, there is also a heightened possibility of facing health challenges such as a previous diagnosis of tubal cancer. As women age, their risk for certain types of cancers increases, including those affecting the reproductive system. This history of cancer can complicate family planning and pregnancy, as fertility may be impacted by both the cancer and its treatments.

### 3.6. Exceptional Scenarios

Although tubal carcinoma is a rare disease, it may be encountered in clinical practice, and it is therefore important to be able to recognize it and to be up to date with the management of these patients. To this end, we report two clinical cases from our clinical practice: a case of incidental STIC with the occurrence of peritoneal carcinoma years later and a case of primary tubal cancer. 

#### 3.6.1. Case 1

We present the case of a 60-year-old woman who underwent prophylactic adnexectomy for BRCA 2 mutation. The patient was tested, after genetic counseling, because of a family history of breast cancer in her mother, maternal aunt and paternal cousin. Prior to surgery, the patient had undergone a six-monthly surveillance protocol with pelvic ultrasound and CA125 combined with Eco mammary and breast examinations every six months and annual mammography and breast magnetic resonance.

She was in good clinical condition, nonsmoking, with a body mass index (BMI) of 26.7 and had been in physiological menopause since the age of 47. The patient’s personal medical history revealed hypertension on medication, and no previous abdominal surgery.

**Figure 2:**
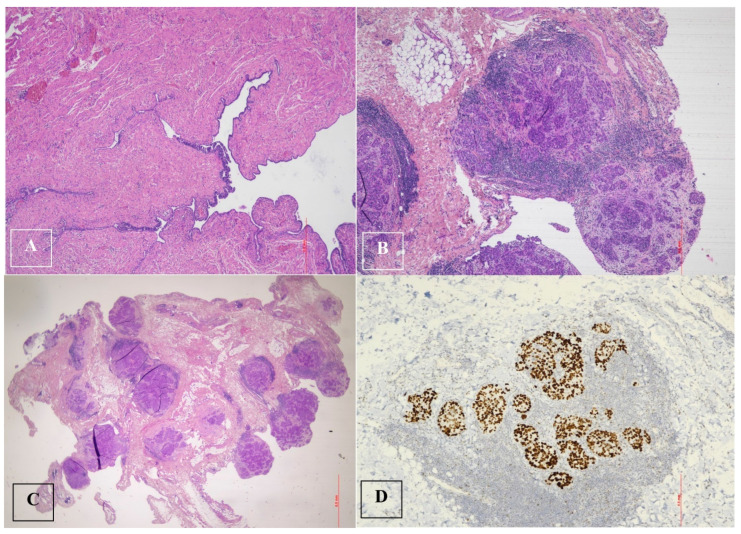
Serous tubal intraepithelial carcinoma (STIC) and high-grade serous carcinoma (HGSC). (**A**). Serous tubal intraepithelial carcinoma (STIC) with irregular luminal surface, and stratified epithelium (contrast with normal mucosa). (**B**,**C**). High-grade serous carcinoma (HGSC) invades omentum (peritoneal) and displays solid architecture. (**D**). p53 staining in peritoneal metastasis: strong and diffuse expression in tumor cell nuclei (>90%), corresponding to p53 missense mutation.

Histological examination after adnexectomy of the distal part of the right salpinx showed the presence of focal papillary proliferation with a multilayered epithelium with marked cytological and immunophenotypic atypia characterized by p53+Wt−/+. The proliferative index, assessed with KI 67, was equal to 60% of cellularity, showing no signs of clear invasiveness; however, the presence of discoid cells in the tubal lumen was consistent with the diagnosis of STIC serous intraepithelial tubal carcinoma (Figure 2). 

No documentable neoplastic proliferation in the left adnexa was detected. The first post-operative transvaginal ultrasound scan showed a regular pelvis with a normal uterus and endometrium, and adnexal fields without any detectable tumefactions.

The case was discussed by the multidisciplinary oncology board and the patient was referred for follow-up and underwent six-monthly mammography and gynecological check-ups with ovarian tumor markers. Due to a senological indication for prophylactic purposes, the patient underwent a bilateral mastectomy one year after the pelvic surgery. All follow-up tests were negative for the next 4 years. 

Four years later, the serum CA125 level was slightly elevated at 46 U/mL. The simultaneous transvaginal ultrasound examination showed a normal pelvis following bilateral adnexectomy, but with minimal fluid collection in the adnexal field of 15 mm on the right side and 20 mm on the left side. 

Less than one month later, CA125 was elevated tobe 72 U/mL and a CT scan was requested. The CT showed a pelvic layer of endoperitoneal fluid between the intestinal loops and in the right inferior parietocolic area.

In the hypogastric region, several mesenteric lymph nodes were noted, the largest of which had a maximum diameter of approximately 9 mm and in the mesogastric region, and an anterior paracaval lymph node measuring approximately 11 mm was detected.

No lymph node swelling was seen in the retroperitoneal space para-aortic region or along the iliac vessels. 

The colonoscopy was negative. 

Positron emission tomography (PET) with fluorodeoxyglucose (FDG) showed suspicion of lymph node heteroplasticity and peritoneal involvement. 

Diagnostic laparoscopy was therefore indicated and revealed the presence of abundant ascitic effusion, which was sent for cytological examination.

A picture of miliariform disease was described, mainly involving the right hemidiaphragm, the omentum, the parietocolic duct, the colon and cecum, both pelvic infundibula, the vaginal rectus septum and the peritoneum of the uterine bladder. Large excisions of the peritoneum were made for histological evaluation.

A final histological examination revealed metastasis of high-grade serous carcinoma.

Three cycles of chemotherapy were administered according to a carboplatin and paclitaxel scheme. CA125 levels decreased from 1337 u/mL before the first cycle to 295 before the third cycle.

The CT of the chest and abdomen performed after the third cycle of chemotherapy showed peritoneal carcinomatosis that had progressed compared to the previous control. The patient underwent a tentative interval debulking surgery. The preliminary laparoscopic view showed that the diaphragm was bilaterally affected by plaque carcinomatosis, sparse carcinomatous nodules at the level of the greater curvature of the stomach, and small omentum, several intestinal loops and mesentery affected by carcinomatosis. Omental cake and peritoneal carcinomatosis were described. 

The pelvis appeared frozen and inaccessible, with the uterus firmly attached to the wall of the sigmoid colon. Because of the impossibility of cytoreduction based on the laparoscopic predictive model for optimal cytoreduction known as the “Fagotti score” [35], extensive biopsies were performed.

A histological examination revealed multiple neoplastic nodules of carcinomatosis infiltrating the tissue with a minimal fibroinflammatory reaction and areas of necrosis.

#### 3.6.2. Case 2

We present the case of a 72-year-old woman with an incidental finding at TVS of an adnexal cyst. The family history was positive for endometrial cancer and gastrointestinal tumors, while patient anamnesis was positive only for hypertension. She had a history of abdominal surgery for appendicitis, cesarean section and laparotomy for diverticulitis more than 30 years ago. 

A pelvic mass was found during a routine gynecological examination. 

The tumor marker CA 125 was negative. The patient was asymptomatic and did not report any vaginal discharge, pelvic mass or abdominal pain. 

A contrast MRI confirmed the presence of right tubal dilatation with solid tissue of 27 × 18 mm.

**Figure 3:**
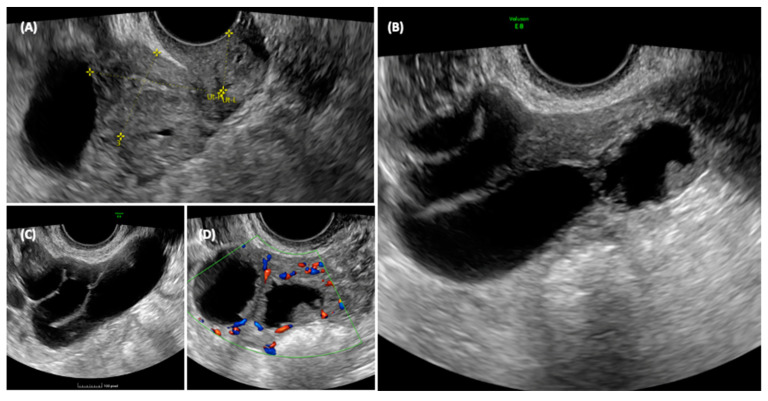
Ultrasonographic appearance of the pelvis. In the transvaginal scan, the anteverted uterus is visible in the longitudinal section (**A**), with a size compatible with the patient’s age. At the fundus of the uterus, a cystic formation is visible, closely adhering to the body of the uterus, and posterior to the uterus, the solid component of the formation can be seen. The detail of the cyst formation is visible in images (**B**,**C**). A unilocular cyst formation with incomplete septa is observed. In image (**B**) it appears that there is tissue protruding into the lumen of the cyst, mimicking the characteristic cog-wheel sign typical of inflammatory pathology, but when images are obtained in different planes (**C**,**D**) it is clear that this is in fact solid tissue, probably of a neoplastic nature, intensely vascularized.

She was sent for an outpatient level II ultrasound. On transvaginal ultrasound, a 70 × 45 mm multilocular elongated cystic formation was described in the right adnexal field, posterior to the uterus, with anechoic content, characterized by irregular internal walls due to the presence of multiple papillae, the largest measuring 15 × 8 mm, vascularized on a color Doppler with a color score of 3. Approximately 3 cm of solid tissue was visualized cranially to this structure, with inhomogeneous content and poorly vascularized on the color Doppler (Figure 3). The ipsilateral ovary was not visualized, while the uterus and contralateral ovary appeared normal. Endometrial polyps were suspected due to the presence of a hyperechogenic structure within the endometrium. 

The ultrasonographic aspect of the pelvis is shown in Figure 3. 

**Figure 4:**
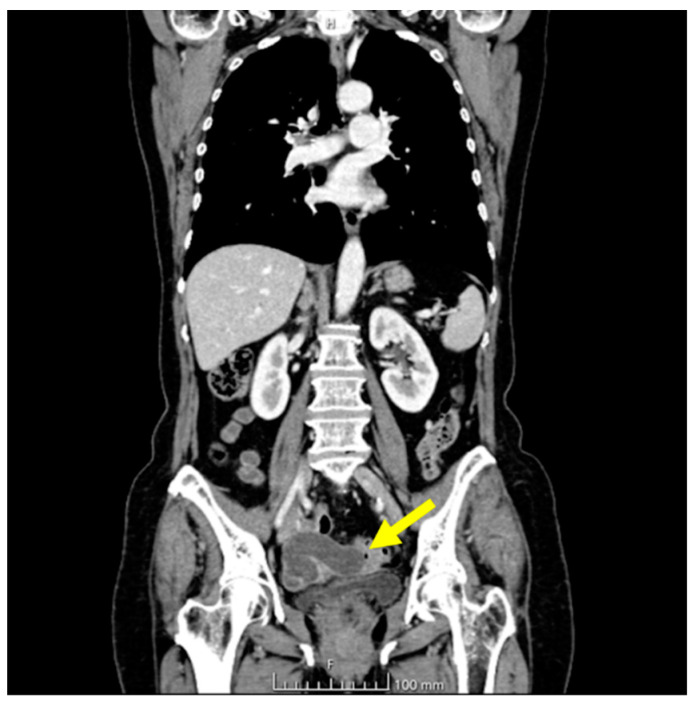
CT chest and abdomen with contrast, portal phase. The yellow arrow indicates the tubal lesion.

The ultrasonographic appearance was considered suspicious for tubal neoplasia and the patient was referred for surgery. Prior to surgery, the patient underwent a CT scan with contrast, which showed the uterus and adnexa with poorly visible cleavage planes, with the presence of a tubular cystic mass compatible with hydrosalpinx. The uterus and adnexa also appeared difficult to separate from the small bowel and sigmoid colon. No ascites or metastatic lesions were present (Figure 4).

An exploratory laparoscopy was performed, showing an upper abdomen with a regular diaphragmatic surface peritoneum, and normal liver, stomach and omentum morphology. Observation of the lower abdomen revealed serpiginous swelling due to an actasic process of the right fallopian tube, about 10 cm in length, adhering tenaciously to the rectus sigmoid and the posterior wall of the uterus, leading to an obstruction of the Douglas. Cerebroid, whitish, friable and frankly neoplastic material was observed oozing from the fimbriated portion. A Fagotti score of 2 was obtained and conversion from laparotomy to cytoreductive surgery was decided upon. The patient underwent bilateral adnexectomy, radical class B1 hysterectomy according to Querleu and Morrow, pelvic peritonectomy of the Douglas and parietocolic ducts, omentectomy and aspiration of peritoneal fluid. An extemporaneous histological examination of the neoformation was requested, which was positive for a carcinomatous epithelial proliferation of tubal origin.

**Figure 5:**
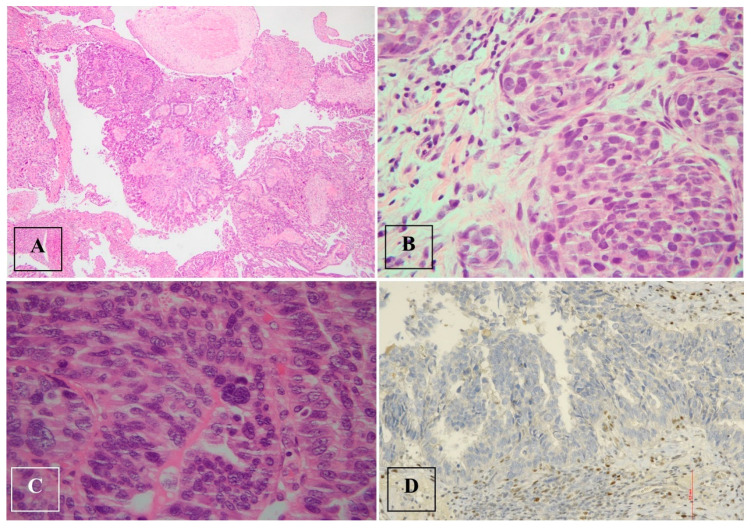
High-grade serous tubal carcinoma. (**A**–**C**). High-grade serous tubal carcinoma with papillary and micropapillary architecture: malignant cells show severe nuclear atypia and significant nuclear pleomorphism with large bizarre and multinucleated form. (**D**). p53 staining in high-grade serous tubal carcinoma: null phenotype.

The final histological examination revealed a high-grade serous carcinoma of the right tuba involving the ipsilateral ovarian surface, parenchyma and adnexal tissues (Figure 5). 

There was a complete absence of expression in the tumor cell nuclei, corresponding to a p53 nonsense mutation (a non-neoplastic stroma serves as an internal positive control).

Neoplastic angiolymphatic permeation was documented. The uterus, omentum and peritoneum were free of carcinomatous infiltration.

This case was discussed at the gynecological oncological multidisciplinary group, genetic counseling was provided, and BRCA1 mutation testing was requested. The patient was then considered eligible for adjuvant chemotherapy.

## 4. Discussion

Fallopian tubes are 11–12 cm long seromuscular structures originating from the uterine horns and extending laterally, connected to the ovary by the mesosalpinx. In the physiological state, the lumen is 1 mm, while the tubular structure constitutes three strata which go from the outside to the inside: the serosa, the muscular layer divided into an inner circular and an outer longitudinal stratum, and the inner mucosa covered by cilia [36]. They are classically divided into four parts, the intramural portion, the isthmus, the ampulla and the infundibulum [36], and receive vascular supply from anastomoses between the ovarian artery and the ascending branches of the uterine artery. 

Inflammatory cytokines resulting from exposure to ovulation and retrograde menstruation may support carcinogenic mutations in the fimbriated portion of the fallopian tube, leading to the occurrence of preneoplastic lesions [11].

As reported above, a strong association has been found between HGSC and serous tubal intraepithelial carcinoma. This association suggests a possible progression from STIC to ovarian and peritoneal HGSC, although the STIC itself is likely to have metastatic potential [6].

A milestone in advancing our understanding of epithelial ovarian carcinogenesis is the work of Kurman [3]. Historically, his group proposed a dualistic model of epithelial ovarian carcinogenesis, dividing lesions into type I and type II tumors. Type I tumors include endometrioid, low-grade serous, clear cell and mucinous carcinoma, while type II tumors comprise high-grade serous carcinoma, carcinosarcoma and undifferentiated carcinoma. Type I neoplasms generally show an indolent nature and are responsible for 10% of deaths due to ovarian cancer, while type II are generally detected in an advanced stage and are highly aggressive, with a massive focus on the mesentery and omentum at diagnosis. Type II tumors account for 90% of deaths from ovarian cancer; moreover, type II neoplasms show chromosomal instability, which is generally not present in type I cancers, and are characterized by TP53 mutations.

This pattern supports the close relationship between tubal preneoplastic lesions and ovarian cancer; hence, the presence of a p53 mutation has been recognized in both entities [5]. Type I carcinomas generally develop from benign or borderline precursors, whereas in type II, the development of malignant lesions is generally de novo, with the only precursor found being STIC [3]. 

Even though STIC is a precancerous lesion confined within the epithelium, it presents cells that are able to spread even without invading the surrounding tissue [3]. The most widely accepted theory is that dissemination is achieved by the detachment of cells from the surface of the fallopian tube [37]. The necessary time for this event’s progression is not yet known, leading to a problem in programming screening; moreover the majority of women at high risk for developing ovarian cancer who received an effective sonographic diagnosis of tubal/ovarian neoplasm had had a previous regular examination 6–12 months before [3].

Because of their proximity to the fimbriated ends of the fallopian tubes, the ovaries are usually the first organ to be affected by the desquamative process of STIC cells and the consequent possible development of ovarian HGSC, although the cells may also directly adhere to the peritoneal surface or omentum to form peritoneal primary HGSC [23].

Visvanathan et al. [37] conducted a multi-center study to comprehensively examine risk and protective factors associated with tubal precancerous lesions in women at high risk of ovarian cancer. They found a prevalence of unique tubal lesions in 6.3% of cases, with no significant differences in BRCA 1 or 2 mutations. Invasive cancer was found in older women, making age the only significant predictor of having a malignant lesion.

Tubal cancer may display several histotypes, the most frequent of which is adenocarcinoma, mainly serous papillary carcinoma (80%), followed by clear cell carcinoma (2%), endometrioid carcinoma (7%) and squamous cell carcinoma, although in most cases, this cannot be clearly distinguished from ovarian cancer [2].

In the majority of cases, 87–97%, lesions are unilateral, with an estimated mean tumor size of 5 cm [2]. Patients usually have a history of vaginal discharge, abnormal uterine bleeding and lower abdominal pain with a palpable pelvic mass or ascites [2]. Latzko’s triad of symptoms is rare (10%) but highly diagnostic of tubal cancer, and is defined by the presence of intermittent colicky pelvic pain, a pelvic mass and bloody aqueous vaginal discharge known as ‘hydropstubae profluence’.

Sherman et al. [10] reported a rate of clinically occult cancer in 2.6% of high-risk patients undergoing bilateral salpingo-oophorectomy. BRCA 1–2 mutations, postmenopausal status, elevated CA125 levels and abnormal findings on transvaginal ultrasound were identified as risk factors in these patients.

There is an open question regarding the relationship between the benefits of RRSO and the potential complications of early surgical menopause. It remains unclear at what age surgery provides the most appropriate protection with minimal adverse effects due to hormone withdrawal. Recent data suggest that salpingo-oophorectomy should be performed between the ages of 30 and 35 years in patients with the BRCA 1 mutation, while it can be delayed to around 40–45 years in patients with the BRCA 2 mutation, hence the idea of performing an early salpingectomy and delaying oophorectomy to a later age to reduce hormonal complications, although this approach is still under investigation.

Follow-up protocols are the only available strategy to detect early disease in high-risk patients. Despite this, Sherman et al. [10] found normal CA125 levels and TVS in all patients diagnosed with STIC.

In the case of metastases from serous endometrial tumors, lesions resembling STIC may be observed, which poses a problem for a differential diagnosis. Some authors suggest that in these cases, a careful microscopic examination of the endometrium should be performed in patients undergoing RRSO [10].

An aspect that needs to be taken into account is that women with a known BRCA mutation may experience high levels of psychological distress due to the possible occurrence of cancer and may need psychological support, even though most cancer concerns seem to recede after RRSO [19].

The current literature review, including the works selected through the search string on PubMed, showed that tubal carcinoma is often classified under the term “ovarian cancer” both in clinical trials and in guidelines. From a clinical perspective, in terms of surgical therapy, neoadjuvant chemotherapy in cases of advanced disease, adjuvant chemotherapy in cases of early disease, and follow-up, the entities are indeed similar. However, it is necessary to consider the distinction between the two entities in the diagnostic phase and during the preoperative radiological staging. Primary tubal carcinoma, in fact, shares the characteristic with ovarian carcinoma of being definitively diagnosed only after a surgical procedure that involves the removal of the lesion and subsequent histological examination. During the diagnostic suspicion phase, tubal carcinoma is differentially diagnosed with various pelvic diseases, both tubal and extra-tubal (such as intestinal, peritoneal and retroperitoneal diseases), both benign and malignant. Therefore, it is crucial to consider the clinical entity of tubal carcinoma itself to avoid a diagnostic delay and, consequently, a therapeutic delay that could negatively impact the patient’s prognosis.

The 11 studies selected from the search string reveal that recent clinical trials have focused on the role of RRSO in women carrying BRCA gene mutations [10], on the possibility of diagnostic radiological/biochemical screening to prevent or provide an early diagnosis of tubal/ovarian cancer [11,13,18], on the role of CT in the preoperative staging of advanced ovarian/tubal diseases [16], on the identification of risk and protective factors for tubal/ovarian neoplasms to target high-risk populations to control the disease through closer preventive clinical surveillance [11,13,17], and on the comparison between PDS and IDS in the surgical therapy of advanced-stage diseases [12,14,15]. The datasets from which these data arise are often the same [12,15,16] or come from other research projects that have published preliminary and definitive data [13,17,20].

As highlighted in specific columns of Table 1, the percentage of tubal/ovarian patients undergoing PDS and IDS varies widely depending on the patient populations, the protocol implemented in each Center, and the expertise of the surgeon managing tubal/ovarian disease. To homogenize this trend, it would be crucial to standardize intraoperative surgical staging and utilize validated, reproducible and well-acquired tools, such as the so-called Fagotti score (laparoscopic predictive model for optimal cytoreduction in advanced ovarian carcinoma) [34,35] and the Vizzielli score (laparoscopic risk-adjusted model to predict major complications after primary debulking surgery in ovarian cancer) [38].

## 5. Conclusions

The cornerstone of tubal tumor treatment is optimal cytoreduction with the surgical removal of all of the disease to ensure the absence of any macroscopic residual tumor, and possibly adjuvant chemotherapy according to the FIGO staging [8]. Obtaining a correct radiological differential diagnosis in the initial diagnostic suspicion phase in order to promptly refer a patient with tubal cancer to the appropriate referral center is essential to ensure the best possible prognosis for this patient. Having a dedicated surgical team and an expert pathology anatomy service for the differential diagnosis of neoplastic and preneoplastic lesions of the fallopian tubes is necessary. 

This review demonstrates that the multidisciplinary team managing oncological gynecological patients must have a complete understanding of the pathophysiology, biology and natural history of the tubal neoplasms to provide adequate treatment.

## Figures and Tables

**Table 1:** Data collection.

First Author, Year, Title [Ref]	Country, Duration of Observation	Type of Study	Aim of the Study	Tubal/ Ovarian/ Peritoneal Cancer Patients	Primary Tubal Cancer Patients N (%)	PDS N (%)	IDS N (%)
Sherman, 2014 [10] Pathologic Findings at Risk-Reducing Salpingo-Oophorectomy: Primary Results From Gynecologic Oncology Group Trial GOG-0199	United States and Australia, from June 2003 to November 2006	Prospective Trial Gynecologic Oncology Group Protocol-0199 (GOG-0199), the National Ovarian Cancer Prevention and Early Detection Study	This trial looked at detecting tubal/ovarian/peritoneal cancer during risk-reducing salpingo-oophorectomy (RRSO). A total of 2605 high-risk women enrolled in the GOG-0199 trial, with 966 women undergoing RRSO to assess cancer prevalence at the baseline surgery.	25	10 (40%)	25 100%)	0
Terada, 2016 [11] Differences in risk for type 1 and type 2 ovarian cancer in a large cancer screening trial	United States, from November 1993 to July 2001	Prospective trial Prostate, Lung, Colorectal, and Ovarian (PLCO) cancer screening trial	This study investigated the impact of previous gynecologic surgery, hormone use and non-steroidal anti-inflammatory drugs on the risk of type 1 and type 2 ovarian cancer (OC). Data from the Prostate, Lung, Colorectal, and Ovarian (PLCO) cancer screening trial were utilized, dividing OC into three groups. Ibuprofen use was linked to a decreased risk of type 1 OC, while tubal ligation, oral contraceptive use and a history of ectopic pregnancy were associated with decreased risks of type 2 OC. The findings suggested that the fallopian tube plays a significant role in carcinogenesis for both OC types.	486	-	-	-
Onda, 2016 [12] Comparison of treatment invasiveness between upfront debulking surgery versus interval debulking surgery following neoadjuvant chemotherapy for stage III/IV ovarian, tubal, and peritoneal cancers in a phase III randomised trial: Japan Clinical Oncology Group Study JCOG0602	Japan, from November 2006 to October 2011	Phase III prospective randomised trial Japan Clinical Oncology Group Study JCOG0602	This trial compared upfront primary debulking surgery (PDS) and interval debulking surgery (IDS) after neoadjuvant chemotherapy (NACT) for stage III/IV ovarian/tubal/peritoneal cancers. The findings indicated that NACT treatment is less invasive than standard treatment.	301	5 (1.6%)	149 (49.5%)	152 (50.5%)
Gentry-Maharaj, 2017 [13] Changing trends in reproductive/lifestyle factors in UK women: descriptive study within the UK Collaborative Trial of Ovarian Cancer Screening (UKCTOCS)	United Kingdom, from April 2001 to October 2006	Prospective birth cohort analysis UK Collaborative Trial of Ovarian Cancer Screening (UKCTOCS)	In this trial, with 202,638 postmenopausal women recruited, differences in reproductive factors were registered across UK birth cohorts. Younger cohorts reported a lower age of menarche, a smaller family size, and increased use of oral contraceptives and infertility treatments, along with a decrease in menopause age post-1945. These shifts in hormone exposure may contribute to trends in breast, endometrial and ovarian cancers, osteoporosis, heart disease and neurodegenerative disorders. Further study could clarify their impact on disease incidence and mortality in detail.	-	-	-	-
Rouzier, 2017 [14] Efficacy and safety of bevacizumab-containing neoadjuvant therapy followed by interval debulking surgery in advanced ovarian cancer: Results from the ANTHALYA trial	France, from January 2013 to June 2014	Prospective phase II study ANTHALYA trial	This study compared two neoadjuvant chemotherapeutic regimens, carboplatin-paclitaxel (CP) vs. bevacizumab-carboplatin-paclitaxel (BCP), for patients with initially unresectable stage IIIC/IV ovarian, tubal, or peritoneal cancer. The results showed that the complete response rate (CRR) with BCP was significantly higher than the reference rate. This study suggests that adding bevacizumab to the preoperative program for non-optimally resectable patients may be safe and beneficial, regardless of the final surgical decision.	205	-	71 (34.6%)	134 (65.4%)
Onda, 2020 [15] Comparison of survival between primary debulking surgery and neoadjuvant chemotherapy for stage III/IV ovarian, tubal and peritoneal cancers in phase III randomised trial	Japan, from November 2006 to October 2011	Phase III prospective randomised trial Japan Clinical Oncology Group Study JCOG0602	This study investigated the comparison between primary debulking surgery (PDS) and neoadjuvant chemotherapy (NACT) for stage III/IV ovarian, tubal, and peritoneal cancers. The EORTC55971, the CHORUS study and the preliminary analysis published by Onda et al. in 2016 showed that NACT was noninferior to PDS. However, a final analysis, including overall survival (OS) as the primary endpoint, did not confirm the noninferiority of NACT. This study suggests that NACT may not always be a substitute for PDS, but due to the smaller sample size, the findings of previous studies supporting NACT’s noninferiority cannot be dismissed.	301	5 (1.6%)	149 (49.5%)	152 (50.5%)
Onda, 2021 [16] Stage III disease of ovarian, tubal and peritoneal cancers can be accurately diagnosed with pre-operative CT. Japan Clinical Oncology Group Study JCOG0602	Japan, from November 2006 to October 2011	Phase III prospective randomised trial Japan Clinical Oncology Group Study JCOG0602	This study compared computed tomography (CT) staging with surgico-pathological staging in advanced ovarian cancer patients undergoing neoadjuvant chemotherapy (NACT). CT staging showed high accuracy for identifying surgical stage III disease but was less reliable for specific details like small extra-pelvic peritoneal disease. While CT staging can be a reliable surrogate for diagnosing stage III disease without surgical diagnosis, its reliability for diagnosing stage IIIB disease (lesions smaller than 2 cm) is inadequate.	301	5 (1.6%)	149 (49.5%)	152 (50.5%)
Taylor 2021 [17] Association of hysterectomy and invasive epithelial ovarian and tubal cancer: a cohort study within UKCTOCS	United Kingdom, from 2001 to 2005, follow-up until December 2014	Prospective study UK Collaborative Trial of Ovarian Cancer Screening (UKCTOCS)	This study investigated, through questionnaires, 202,506 postmenopausal women from the UK Collaborative Trial of Ovarian Cancer Screening (UKCTOCS). It explored if hysterectomy with conservation of the adnexa affected ovarian/tubal cancer risk. The results showed that 0.55% of women with hysterectomy and 0.59% with intact uteri were diagnosed with ovarian/tubal cancer, indicating no significant association. This study reinforces that hysterectomy does not alter invasive ovarian and tubal cancer risk. These findings are crucial for clinical counseling and improving risk prediction models.	1176 (178 type I, 890 type II, 108 type uncertain)	-	-	-
Maurer, 2022 [18] Randomised controlled trial testing the feasibility of an exercise and nutrition intervention for patients with ovarian cancer during and after first-line chemotherapy (BENITA-study)	Germany, from April 2018 to Sept 2019	Randomized controlled Trial The BENITA (Bewegungs- und Ernährungsintervention bei Ovarialkrebs) study	This pilot study evaluated a combined exercise and nutrition intervention’s safety and acceptance during and after first-line chemotherapy for advanced ovarian cancer following primary or interval debulking surgery. This study, conducted as a randomized controlled trial (RCT), demonstrated the intervention’s safety and acceptance. The larger BENITA study aims to investigate the intervention’s impact on quality of life, fatigue and survival, with plans to integrate it into oncology guidelines and clinical practice.	15	-	12	3
vanBommel, 2022 [19] Cancer worry among BRCA1/2 pathogenic variant carriers choosing surgery to prevent tubal/ovarian cancer: course over time and associated factors	Netherlands, from 2015 to present	Prospective study Prospective TUBA-study (NCT02321228): Early Salpingectomy (Tubectomy) With Delayed Oophorectomy in BRCA1/2 Gene Mutation Carriers (TUBA)	This study evaluated 577 BRCA1/2-PV carriers: 57% had high cancer worry pre-surgery, decreasing to 54% post-surgery. Factors influencing high worry were age, unemployment, prior breast cancer, lower education and recent diagnosis. While most saw decreased worry after surgery, a subset (6%) maintained major concerns even a year later, suggesting the need for extra support for this group.	-	-	-	-
Menon, 2023 [20] Mortality impact, risks, and benefits of general population screening for ovarian cancer: the UKCTOCS randomised controlled trial	United Kingdom: 27 primary care trusts adjacent to 13 trial centers based at NHS Trusts in England, Wales and Northern Ireland, from April 2001 to September 2005, screening until December 2011, follow-up until 2020.	Randomized controlled trial. UK Collaborative Trial of Ovarian Cancer Screening (UKCTOCS)	This study compared two screening methods, multimodal screening (MMS) and ultrasound screening (USS), with a control group receiving no screening. Postmenopausal women aged 50–74 with intact ovaries and no history of ovarian or non-ovarian cancer were divided into three groups. Over a 16.3-year follow-up, both MMS and USS did not show a significant reduction in deaths due to ovarian or tubal cancer compared to the control group. The MMS group had higher rates of detecting early-stage cancer, while the USS group did not show a difference in cancer stage detection compared to the control group.	2055 (1% of all enrolled women) 522 of 50,625 in the blood group 517 of 50,623 in the scan group 1016 of 101,314 in the no-screening group	-	-	-

PDS: primary debulking surgery; IDS: interval debulking surgery.

**Table 2:** Tubal precursor lesions.

Precursor Lesions	Epidemiology	Clinical Presentation	Diagnostic Aspects Hematoxylin and Eosin	Immunohistochemistry	Evolution
STIC Serous tubal intraepithelial carcinoma	Found in 4% of tubes in patients undergoing salpingectomy for non-neoplastic indications	Usually asymptomatic, occasional diagnosis during salpingectomy or salpingo-oophorectomy	* -Irregular luminal surface -Epithelial stratification -Cellular or nuclear pleomorphism -Nuclear enlargement -Nuclear hyperchromasia -Mitotic figures, prominent nucleoli and/or apoptotic bodies	p53 mutant type and a high Ki-67/MIB-1 index (≥10%)	HGSC ovarian carcinoma in 10% Time to carcinoma progression: 7 years
STIL Serous intraepithelial lesion of the fallopian tube	Found in 9% of tubes in patients undergoing salpingectomy for non-neoplastic indications	Usually asymptomatic, occasional diagnosis during salpingectomy or salpingo-oophorectomy	Resembles STIC but shows less than three features required for STIC diagnosis on hematoxylin and eosin staining	p53 negative and/or low Ki-67	Possibile “precursor escape”
TILT Tubal intraepithelial lesion in transition	Found in 3.2% of tubes in patients undergoing salpingectomy for non-neoplastic indications	Usually asymptomatic, occasional diagnosis during salpingectomy or salpingo-oophorectomy	Resembles STIC but shows less than three features required for STIC diagnosis on hematoxylin and eosin staining	p53 negative and/or low Ki-67	When diagnosed in isolation, remains unclarified
SCOUT Secretory or stem cell outgrowths	Found in 45% of tubes in patients undergoing salpingectomy for non-neoplastic indications	Usually asymptomatic, occasional diagnosis during salpingectomy or salpingo-oophorectomy	Linear segments with continuous population of ≥30 secretory cells without intervening ciliated cells	Bcl-2 ** positivity in ≥30 cells Not associated with p53 mutation	Although there is no evidence that they are directly related, there is an increased rate in women with serous carcinoma
p53 SIGNATURE	Found in 2% of tubes in patients undergoing salpingectomy for non-neoplastic indications	Usually asymptomatic, occasional diagnosis during salpingectomy or salpingo-oophorectomy	No clear cytomorphological atypia on hematoxylin and eosin staining	Morphologically normal epithelium with aberrant p53 staining pattern in at least 12 adjacent cells	Early event in the pathway to serous carcinoma

* At least 3 of these features on hematoxylin and eosin staining are required to diagnose STIC. ** B-cell lymphoma 2.

## Data Availability

Data supporting the reported results can be requested from the corresponding author.
